# Surprising effects of cascading higher order interactions

**DOI:** 10.1038/s41598-022-23763-z

**Published:** 2022-11-12

**Authors:** Hsun-Yi Hsieh, John Vandermeer, Ivette Perfecto

**Affiliations:** 1 Great Lakes Bioenergy Research Center, Michigan State University, East Lansing, MI USA; 2 Kellogg Biological Station Long-Term Ecological Research, Michigan State University, Hickory Corners, MI USA; 3 Department of Ecology and Evolutionary Biology, University of Michigan, Ann Arbor, MI USA; 4 School for Environment and Sustainability, University of Michigan, Ann Arbor, MI USA; 5 Kellogg Biological Station Long-Term Agroecosystem Research, Michigan State University, Hickory Corners, MI USA

**Keywords:** Ecology, Community ecology

## Abstract

Most species are embedded in multi-interaction networks. Consequently, theories focusing on simple pair-wise interactions cannot predict ecological and/or evolutionary outcomes. This study explores how cascading higher-order interactions (HOIs) would affect the population dynamics of a focal species. Employing a system that involves a myrmecophylic beetle, a parasitic wasp that attacks the beetle, an ant, and a parasitic fly that attacks the ant, the study explores how none, one, and two HOIs affect the parasitism and the sex ratio of the beetle. We conducted mesocosm experiments to examine these HOIs on beetle survival and sex ratio and found that the 1st degree HOI does not change the beetle’s survival rate or sex ratio. However, the 2nd degree HOI significantly reduces the beetle’s survival rate and changes its sex ratio from even to strongly female-biased. We applied Bayes’ theorem to analyze the per capita survival probability of female vs. male beetles and suggested that the unexpected results might arise from complex eco-evolutionary dynamics involved with the 1st and 2nd degree HOIs. Field data suggested the HOIs significantly regulate the sex ratio of the beetle. As the same structure of HOIs appears in other systems, we believe the complexity associated with the 2nd degree HOI would be more common than known and deserve more scientific attention.

## Introduction

Ecological systems are complex systems that frequently involve many interacting species^1^. However, the focus of ecological research over the last 50 years has been mostly on pair-wise interactions. The importance of higher-order interactions (HOIs), here defined as the addition of a third (or multiple) species changing the strength of the pair-wise interaction (i.e. interaction modification, in contrast to interaction chain^2^), has been widely recognized in ecological studies^3–10^. Yet, studies remain in theoretical explorations of large communities focusing on diversity and stability^4,11–13^, or consumption coefficients in small or large ecological communities with only one degree HOIs (i.e. one additional species modifying one pair-wise interaction)^5,7,8,10,14,15^. It is also unclear where HOIs occur in structured food webs^16,17^. Empirical studies involved with communities composed of interacting 1st and 2nd degree HOIs are rare^18–21^. In these studies, the 2nd degree HOI interferes with the 1st degree HOI, negatively impacting the pair-wise interaction. The two HOIs, therefore, interact and produce consequences that are not predictable in additive pair-wise interaction models. Such interactions as results of evolutionary consequences are not described in recent reviews or anthologies, suggesting an open subfield inviting investigations^5,22,23^. A multispecies community in which species interactions are relatively well studied would serve as a vehicle to further scientific understanding of the ecological and evolutionary dynamics concerning higher-order interactions.

**Figure 1:**
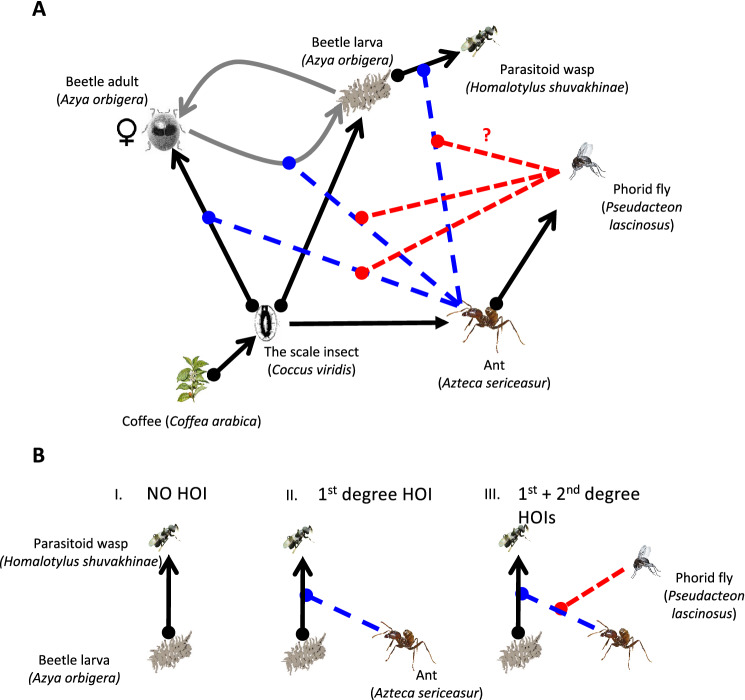
(**A**) The diagram of the dynamics of the study system. The black solid lines with dots (resources) and arrows (consumers) represent trophic relationships. The gray curved arrows represent the growth and reproduction of the beetle. The blue dashed lines represent the 1st degree HOI—the in-discriminant interference of the ant with the beetle and the wasp, and the red dashed lines represent the disruptions of the 1st degree HOI in the presence of the phorid fly, the 2nd degree HOI. The current study aims to understand how the phorid fly would affect the parasitism and the sex ratio of the beetle, noting by the red question mark. (**B**) Diagram of the three types of interactions explored in the experiment. (I) Parasitism of beetle larvae with no HOI; (II) Parasitism of beetle larvae with 1st degree HOI; (III) Parasitism of beetle larvae with the 1st and 2nd degree HOIs (the ant interferes with the wasp and the fly affects ant activity). Black arrows represent direct parasitism, dashed blue lines represent the 1st degree HOI and dashed red line represents the 2nd degree HOI.

Here we explore a subcomponent of a naturally occurring network involving six species, six direct trophic interaction and six HOIs (Fig. 1). This system has been well studied in a neotropical coffee farm in southern Mexico^21,24,25^, but the cascading impact of the HOIs on target organisms is unknown.

At the core of our study system is the mutualism between the ant *Azteca sericeasur* and the green coffee scale, *Coccus viridis* (Fig. 1-A). The mutualism itself depends on an HOI since the ant exchanges nutrition by protecting the scale insects from the coccinellid beetle (*Azya orbigera*) predation, mostly by harassing the beetle adults (Fig. 1-A). The beetle larva is a classic example of myrmecophily, surviving in areas with high ant densities because its waxy filaments protects it from ant predation. The beetle larva is attacked and killed by a parasitic wasp (*Homalotylus shuvakhinae*). Importantly, the ant chases away parasitoid wasps attempting to access the scale insects but is unable to distinguish between scales’ parasitoids and beetle larvae’s parasitoids. Therefore, the ant protects the beetle larvae from parasitoid attacks. The ant is attacked by a phorid fly parasitoid (*Pseudacteon lascinosus*). The phorid fly has a strong HOI effect on the ant^26^. When the phorid is around, the ant adopts a catatonic anti-parasite behavior, significantly lowering ant foraging activity, reducing the strength of the ant interference with other organisms attempting to access the ant-foraging patches^27^.

**Figure 2:**
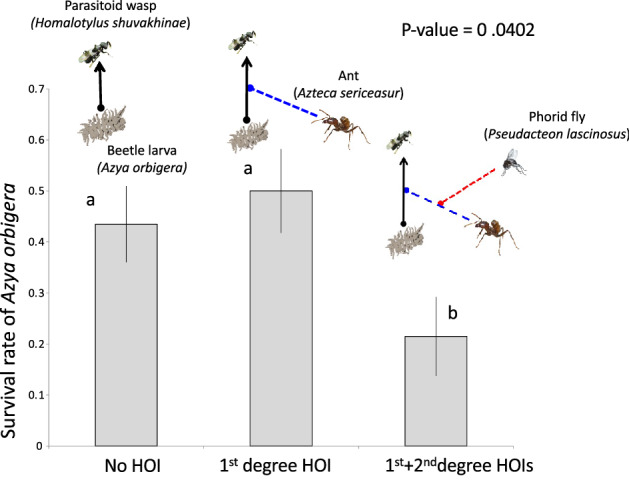
The survival rate of *Azya orbigera* after being exposed to the wasp with no HOI (only the wasp), the 1st degree HOI (the wasp and the ant), and the 1st and the 2nd degree HOIs (the wasp, the ant, and the phorid fly).

The subcomponent of this system in this paper includes: (1) the direct trophic interaction between the parasitoid wasp (*H. shuvakhinae*) and the beetle (*A. orbigera*) (Fig. 1-B-I); (2) the ant interference with the parasitoid wasp (Fig. 1-B-II); and (3) the anti-parasitism behavior of the ant induced by the presence of its parasitoid fly (Fig. 1-B-III).

The catatonic behavior of the ant is chemically mediated and results in the facilitation of the feeding and reproduction of the beetle that preys on the green coffee scale, the mutualistic partner of the ant (Fig. 1-A). The gravid female beetle uses the ant pheromone induced by the phorid to identify the right moment and location to oviposit within patches of high scale densities^21^. However, the male beetle does not appear to be attracted to the ant pheromone at all^21^. Therefore, phorid fly attacks represent an opportunity for gravid female beetles to lay eggs on coffee plants that have ants (*A. sericeasur*) and abundant scale insects, their source of nourishment. Subsequently, the beetle larvae (which are protected from ant predation) enjoy abundant food resources (the scale insect) in the ant-hemipteran patches^21^. Theoretically, the ant acts as the modification species of the consumer-resource interaction (i.e.*,* the 1st degree HOI as it interferes with the feeding and the reproduction of the beetle). The parasitic phorid fly acts as the modification that interferes with the 1st degree HOI (i.e.*,* the 2nd degree HOI that interferes with the 1st HOI). In other words, the effect of the phorid fly (*P. lascinosus*) cascades to the beetle via the interaction of the two HOIs (Fig. 1-A).

In this study, we focus on the effect of the HOI cascade on the survival and the sex ratio of the beetle, two parameters less studied than feeding but of interest to theoreticians exploring non-trophic effects in food webs^28^. Oviparous females seek adequate locations and moments to lay eggs to optimize fitness^29^. The presence of ants and other predators may signal inappropriate habitats for oviparous female insects^30^. However, the reverse is true for myrmecophilous beetles since they benefit from the ants^31,32^. For *A. orbigera*, the presence of *Azteca* ants signals an enemy-free space with plenty hemipterans^21^. Such myrmecophily may be responsible for the observed benefits of ant-hemipteran mutualism in plants^33,34^, and suggests the importance of this mutualism in pest control^25,35^.

In this study, we ask (1) how the phorid fly affects the ant aggressivity toward the parasitoid wasp; (2) how the parasitism rate of the beetle changes under the conditions of no-HOI (only the host-parasitoid interaction is present, Fig. 1-B-I), the 1st degree HOI (the ant interferes with the host-parasitoid interaction, Fig. 1-B-II), and the combination of the 1st and the 2nd degree cascading HOIs (the phorid fly interrupts with ant interference with the parasitoid wasp of the beetle, Fig. 1-B-III); (3) how the conditions of no-HOI, the 1st degree HOI, and the cascading 1st and 2nd HOIs affect the sex ratio of the beetle population surviving from parasitism.

As we were only able to identity beetle’s sexes when the beetles emerged, we employed Bayes’ theorem to explore beetle larvae's sex-differential responses to the HOIs. We discussed how the wasp’s host preference and beetle larvae’s sex-differential responses to parasitism and HOIs cause the differences in the parasitism rate and the sex ratio of the beetle in different combinations of HOIs. We also explored the relevancy of our study to the existing literature.

## Method

### Study site

We conducted laboratory studies at the field site in *Finca Irlanda*, which is a 300-hectare organic shaded coffee farm located at 1100-m altitude, in the municipality of Tapachula, the state of Chiapas in Southern Mexico (92° 20′ 29″ W and 15° 10′ 65″ N). For the laboratory experiments, all organisms were freshly collected from *Finca Irlanda* or reared in the lab from insects collected from the field close by. The lab and field work was performed with a permit from the farm owner the Peters family.

### Ant aggression test

To examine the effect of phorid flies (*P. lascinosus*) on the aggressivity of ants (*A. sericeasur*) towards the parasitoids of the beetle larvae (*A. orbigera*), we conducted an ant aggression test with two treatments: with and without phorids. In the first treatment, a small coffee branch containing two leaves with scale insects (*C. viridis*) and 20 ant workers were both introduced into a one-liter plastic container. This was done to mimic as much as possible field conditions where the ants are tending scale insects. After waiting for at least 15 min for the ants to calm down and start tending the scale insects, one third- or fourth-instar larva of the beetle was introduced*.* In the second treatment, all settings were the same except for the addition of 3–4 phorid flies. Once the two treatments were established, one female parasitoid wasp (*H. shuvakhinae*) was released into each container. During a forty-minute trial, each time that a parasitoid wasp encountered an ant worker, the response of the ant individual was recorded. Ant responses to parasitoids were classified into two categories: (1) the ant ignores the wasp; (2) the ant attacks the wasp. All insects were used for a single replicate and then discarded. A total of four replicates were completed for both the presence and absence of phorids. For each trial, we calculated the proportion of actions (either aggressive or none) by ants when encountering the parasitoid wasp in the treatments with and without phorid flies. We used R^36^ to conduct a two-sample Mann–Whitney U test on the proportion of ant actions.

### Parasitism experiments and analyses

To examine the parasitoid wasp’s host preference and the effect of the 1st degree and the 2nd degree HOIs on the beetle’s parasitism and sex ratio, we conducted a laboratory experiment in insect tents (60 cm × 60 cm × 60 cm) with three treatments: (1) no ants (no HOIs but only the wasp and the beetle larvae), (2) ants (1st degree HOI), and (3) ants and phorids (1st and 2nd degree HOIs) (Fig. 1-B). We randomly assigned insect tents to each treatment in each trial, and the tents for each treatment were also shuffled in each trial. All beetle larvae used for these experiments were reared in the laboratory for at least two generations from freshly collected beetle adults. In each tent we placed a coffee branch with 4–6 leaves infested with approximately 100 scale insects inside a plastic container at the center of an insect tent. The set up for the three treatments of species combinations were as follows: (1) 4–5 third or fourth instar beetle larvae and a parasitoid wasp; (2) 4–5 third or fourth instar beetle larvae, a parasitoid wasp, and about 60–80 ant workers; (3) 4–5 third or fourth instar beetle larvae, a parasitoid wasp, about 60–80 ant workers and 3–4 phorid flies. Organism densities in these treatments were close to those observed in the field. To allow for acclimation, we introduced organisms into the tents in the following order: first, we introduced the coffee branch containing scales, immediately followed by the ants (in treatments 2 and 3). After the ants settled down and started tending the scale insects, we introduced the beetle larvae. Once the larvae began moving on the coffee leaves, we introduced the phorids (in treatment 3). When the three treatments were established, and the organisms exhibit normal behavior, we released one lab-reared female parasitoid wasp (*H. shuvakhinae*) in each tent (treatments 1, 2, and 3). We allowed the organisms to interact for 24 h. After 24 h, we collected all beetle larvae in each treatment and reared them with sufficient scale insects as food, until beetle adults emerged or parasitism symptoms appeared (parasitized larvae turned into hardened black mummies). The treatments of no HOI and 1st + 2nd degree HOI were repeated for 10 consecutive times, and the treatment of 1st degree HOI was repeated for 11 consecutive times, with new individuals of each organism. We recorded parasitism instances and beetle sexes upon emergence. To estimate the sex ratio without parasitoid influence, 78 randomly selected beetle individuals were reared on coffee leaves with scale insects without any interaction with other organisms.

To analyze the effect of the parasitoid, the ant and the phorid fly on the parasitism rate and the sex ratio of the beetle, we developed a nested model, starting from

$$logit\left(\widehat{P}(S)\right)=a+bA$$

where $\widehat{P}(S)$ is the probability of an individual being parasitized, *A* is a binary variable, standing for the absence (0) and presence (1) of ants, *a* is the baseline probability of parasitism, and *b* is the magnitude of parasitism altered by ants in the logistic function. We further hypothesized that phorid attacks modify the strength of the interaction modification that ants exert upon the host-parasitoid interaction. Therefore,

$$b=g+hP$$

where *P* is another binary variable, standing for the presence (1) and absence (0) of phorids. Substituting *b,* we obtain the following function,

$$logit\left(\widehat{P}(S)\right)=a+gA+hAP$$

where *g* represents the effect of ants on the parasitism rate of *A. orbigera* larvae, and *h* represents the effect of the fly’s facilitation, via interfering with the ant’s interference on the parasitism rate of *A. orbigera* larvae. We used binary responses (1: survival; 0: parasitized) of all available beetle individuals across the three treatments. We performed model selection based on the Akaike Information Criterion (AIC) and likelihood ratio tests. For the latter, we started model selection by fitting the full model and preceding each step by eliminating the term that had the least significance (the greatest *p-*value) on the explanation of the dependent variable. The analysis was performed with the application of the bbmle package in R. By doing this, we determined the maximum likelihood estimates of survival probability of the beetle, $\widehat{P}(S)$, in the three treatments: (1) *A* = 0, *AP* = 0 (no HOI); (2) *A* = 1, *AP* = 0 (one HOI: ant interference) and (3) *A* = 1, *AP* = 1 (interacting HOIs: phorid interference with ant interference), and errors associated with these estimates.

The same idea applies to the sex ratio of the beetle under the influence of various organisms. We developed the following equation,

$$logit\left(\widehat{P}(F|S)\right)= r+mA+nAP$$

where $\widehat{P}(F|S)$ is the probability of a parasitism survivor being female. *A* and *P* are both binary variables. Respectively, they represent the ant and the phorid fly, and the numeric attributes, 0 and 1, denote their absence and presence. As before, model selection and parameter estimates were conducted with AIC. By doing this, we determined $\widehat{P}(F|S)$, the estimate of being a female beetle given survival, for the three treatments: (1) *A* = 0, *AP* = 0 (no HOI); (2) *A* = 1, *AP* = 0 (one HOI: ant interference) and (3) *A* = 1, *AP* = 1 (interacting HOIs: phorid interference with ant interference), and errors associated with these estimates. We employed the *mle2* function in the bbmle package in R to estimate the female probability (1) in the absence of HOI (the beetle and the parasitoid alone), (2) in the presence of the 1st degree HOI (the beetle, the parasitoid and the ant), and (3) in the presence of the 1st and the 2nd degree HOIs (the beetle, the parasitoid, the ant and the phorid fly).

### Probabilities of per capita female and per capita male survival from parasitism under the influence of ant and the phorid fly

To test whether the sex ratio of beetle survivors’ population is due to sex-differential survival probability, Bayes’ theorem was employed. *Per capita* female survival probability from parasitism in each treatment of the parasitism experiment was derived based on $\widehat{P}(F)$, $\widehat{P}\left(F|S\right),$ and $\widehat{P}(S)$, and *per capita* male survival probability was derived based on $\widehat{P}(M)$, $\widehat{P}\left(M|S\right),$ and $\widehat{P}(S)$. According to the Central Limit Theorem, the estimates of proportions, $\widehat{P}\left(S|F\right)$ and $\widehat{P}\left(S|M\right)$, are approximately normally distributed,

$$\widehat{P}\left(S|F\right)\sim N\left(\widehat{P}\left(S|F\right), \sqrt{\frac{\widehat{P}(S|F)\times \left(1-\widehat{P}\left(S|F\right)\right)}{{n}^{*}}}\right)$$

$$\widehat{P}\left(S|M\right)\sim N\left(\widehat{P}\left(S|M\right), \sqrt{\frac{\widehat{P}(S|M)\times \left(1-\widehat{P}\left(S|M\right)\right)}{{n}^{*}}}\right)$$

with means $\widehat{P}\left(S|F\right)$ and $\widehat{P}(S|M)$, and standard deviations $\sqrt{\frac{\widehat{P}\left(S|F\right)\times (1-\widehat{P}\left(S|F\right))}{{n}^{*}}}$ and $\sqrt{\frac{\widehat{P}\left(S|M\right)\times (1-\widehat{P}\left(S|M\right))}{{n}^{*}}}$, where $\widehat{P}(S|F)$ and $\widehat{P}(S|M)$, respectively, are the population proportions of females and males. Here we employ n*, the smallest sample size among those of the three variables in the Bayesian formulas for males and females. Since the three variables have different sample sizes, n* guarantees a conservative estimate of standard error, and thus confidence interval, of each derived probability.

## Results

### Ant aggression test

In general, ants behave aggressively against the parasitoid wasps. However, the number of ant attacks on parasitoids was significantly lower in the presence of phorid flies, declining from 76.25 ± 9.47% without phorids to 43.75 ± 6.41% with phorids (*p*-value = 0.028; Fig. S1 in Supplementary Materials).

### Survival and sex ratio of the beetle

*Azya orbigera* survival rate from parasitism in the treatment with no HOIs (i.e., no ants) was 43.06 ± 7.2%. The survival rate of beetles in the treatment with ants (the 1st degree HOI) was higher but not statistically significantly different from the survival rate without ants (Fig. 2). However, the survival rate of beetles exposed to ants (*A. sericeasur*) and phorid flies (the 1st + 2nd degree HOIs) declined significantly to 20.41 ± 5.82% (Fig. 2).

**Figure 3:**
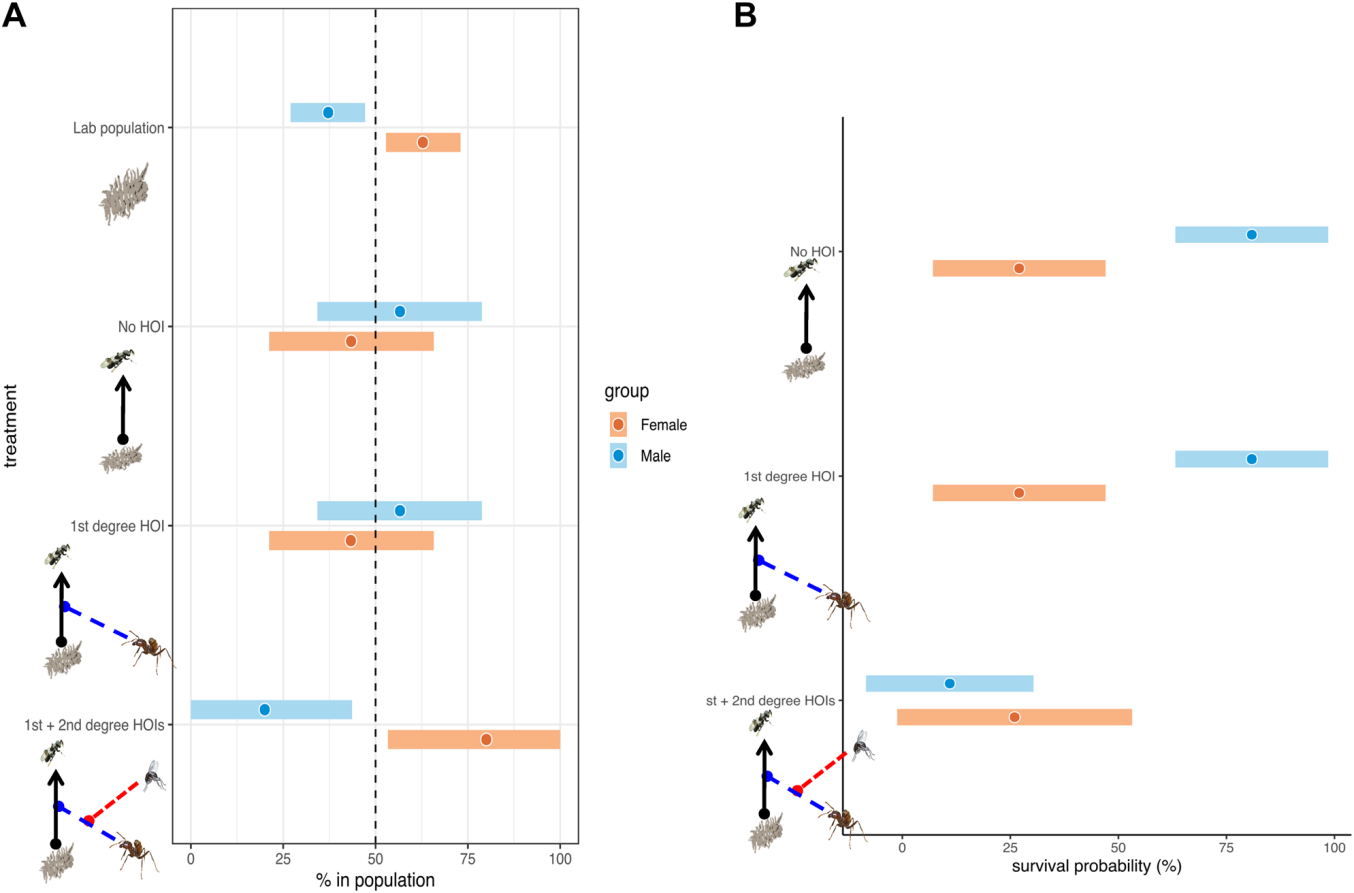
(**A**) Female and male ratios in four different conditions, lab population, no HOI (only beetle larvae + wasps), the 1st degree HOI and two interacting HOIs. The dots in the bars are the means and the extends of the bars are the 95% confidence intervals. (**B**) The per-capita survival probability of the female ($\widehat{P}\left(F|S\right)$) and male beetles ($\widehat{P}(M|S)$) is computed by Bayes’ Theorem. The result shows that the parasitism probability of the female remains the same across the treatments, while the presence of the phorid fly significantly reduces the survival probability of the male.

Concerning the sex ratio, the lab-reared population was female-biased with 62.82 ± 5.51% female beetles. Adding parasitoid wasp altered the female-biased beetle population to be sex-balanced (female ratio = 43.48 ± 11.37%), suggesting that the parasitoid wasps, *H. shuvakhinae*, preferentially attack female beetles. However, adding the ants (*A. sericeasur*) (the 1st degree HOI) did not affect the sex ratio of the beetle survivors’ population. On the other hand, the phorid fly’s interference with ant harassment (the 1st + 2nd degree HOIs) reversed the sex ratio of the beetle to be strongly female-biased (female ratio = 80 ± 12.06%) (Fig. 3A).

Analyzing the derived *per capita* male and the *per capita* female beetle survival probabilities showed a higher survival probability for males when there is no HOIs, and when there is the 1st degree HOI. However, the *per capita* male survival probability declines dramatically in the presence of phorids. On the other hand, the *per capita* female beetle survival probability remains constant across the three treatments (Fig. 3B).

## Discussions

Recent ecological literature has emphasized that HOIs have profound impacts on ecological systems. Reviews and empirical studies suggest that, by initiating the phenotypic response of a resource or a competitor, a modification species can cause strong effects on a pair-wise interaction, consequently leading to strong effects at population, community, and ecosystem levels^5,7,37^. Theoretical and empirical studies found that HOIs increase the robustness of food webs^4,11–13^, and the structure of two interactive HOIs can stabilize biodiversity-rich ecological networks^11^. With such a recognition, however, most theoretical and empirical HOIs studies are limited to answer how a pairwise interaction is affected by a third species^5,14,15^. Predicting the effects of HOIs in multispecies ecological networks is still challenging^28,38,39^. Unknown evolutionary response of interacting organism might contribute to this difficulty.

In our study, we found clear evidence that the ant (*A. sericeasur*) interferes with the parasitic wasp (*H. shuvakhinae*), and that the presence of the phorid fly (*P. lascinosus*) reduces the strength of this interference (Fig. S1), likely facilitating the parasitism of the beetle (*A. orbigera*). Given these results, the expectation was that the presence of the 1st degree HOI (the ant), would increase the beetle survival, while the addition of the 2nd degree HOI (the phorid flies) would reduce that effect. Instead, our results show that, although the survival rate of the beetle increased slightly with the introduction of the ants (the 1st degree HOI), that effect was not statistically significant. However, the introduction of the phorids (the 2nd degree HOI) significantly decreased the survival rate of the beetle (Fig. 2). Given the strong evidence that the ants do behave aggressively toward and interfere with the parasitic wasps, it is puzzling that we did not detect a significant positive effect of the ants on the survival rate of the beetle. A possible explanation for this result is that the wasps preferentially attack beetle larvae that are associated with the ants. This could be an evolutionary response since beetle larvae in areas with ants tend to have more food available since the ants protect the scale insects that the beetle larvae eat. Furthermore, olfactometer experiments with adult beetles show that female adult beetles are attracted to pheromones released by this ant species^21^. It is possible that the wasp uses visual or chemical cues of the ant to locate their beetle host. If this is happening in this system, that means that the treatments with ants (1st degree HOI and the 1st + 2nd degree HOI) stimulate higher parasitoid attacks to the beetle, but, since the ants behave aggressively towards the parasitoid wasps, this effect is cancelled resulting in no significant increase in beetle survival. This explanation also fits with the result that the survival rate of the beetle in the 1st + 2nd degree HOI treatment (ants + phorid flies), is much lower than the survival rate of the no HOI treatment, since the latter has no ants and therefore provides less stimulus for the parasitoid to search, find and attack the beetle larvae.

**Figure 4:**
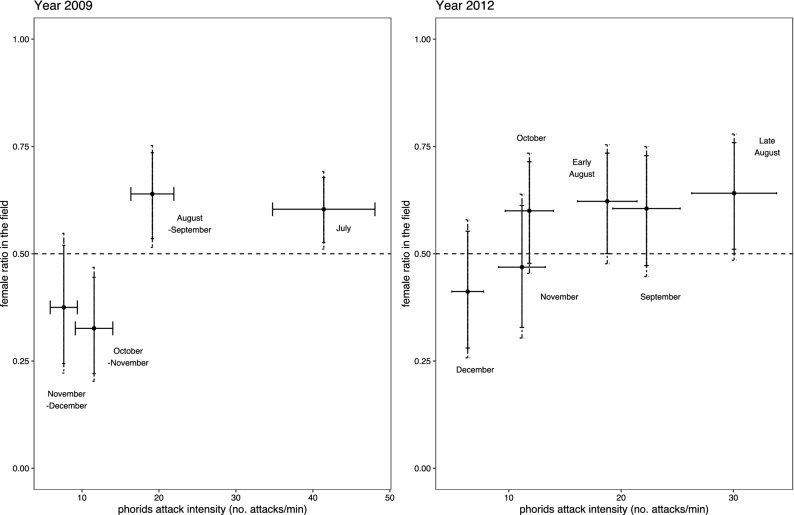
Relationship between phorid attack intensity (attack counts/min) and female ratio of *A. orbigera* in multiple surveys in the 45-hectare permanent plot of *Finca* Irlanda in 2009 and 2012. Cross centers represent mean values, solid and dashed lines, respectively, *along* the y-*axes*, representing the 90% and 95% confidence intervals of female ratios, and errors on the *x*-axes represent the standard errors of phorid attack intensities.

Our study also found that HOI have important and significant effects on the sex ratio of the beetle. A laboratory reared population of the beetle with no interference with other organisms demonstrated that beetle populations tend to be female biased. The wasp appears to favor female beetle hosts as it caused the female-biased beetle population to be sex balanced (Fig. 3A). Although wasp’s host preference and ant interference may explain the unchanged female ratio in the 1st degree HOI treatment, the window of opportunity opened up by the phorid (the 2nd degree HOI) counterintuitively results in a female-biased beetle population (Fig. 3A). To explore such discrepancy, we employ Bayes’ theorem to derive the per capita female vs. per capita male survival probability in each combination of HOIs (no HOI, the 1st degree HOI, and the 1st and the 2nd degree HOIs). We discover that the survival probability of the female beetle remains unchanged in these treatments. However, the survival probability of the male beetle is significantly reduced when the 1st and the 2nd degree HOIs both exist (Fig. 3B). The difference in sex ratio between the lab-reared beetle population with no interactions and the same population with wasp parasitism (Fig. 3A) supports the argument that the male beetle is unfavored by the wasp. But what emerges from examining the per capita survival rate of male and female beetles is the apparent resistance of the favored female beetle to parasitism (Fig. 3B). The mechanism for such resistance is not known but it could be that the female beetle larvae are spatially more tightly associated with ants and even under phorid attack, the ants managed to offer some protection to those beetles that are very close to them (I.P. personal observation). Male beetle larvae, on the other hand, are randomly distributed and therefore have less protection from the ants even when these are being attacked by the phorid flies. This interpretation of the results is supported by field surveys of beetle adults and phorid attack intensity on the ant in a 45-hectare permanent plot in *Finca Irlanda* (Fig. 4) (Methods in Supplementary Materials). These surveys show that the beetle population is female-biased when the phorid attack intensity is high in early rainy season (July–August). The population becomes sex-balanced or even male-biased when the phorid attack intensity on the ant declines. Although we cannot tell apart how much of the consequence of beetle’s female ratio in the ant patches comes from female adults’ attraction to the ant^21^, and how much it comes from the complex parasitoid wasp—larvae—HOIs effects, the result does suggest that the phorid-ant interaction has an effect on the sex ratio of the beetle, making it more female biased.

Combined with our previous studies, we conclude that the phorid fly (1st + 2nd HOIs) can influence the feeding^20^, reproduction^21^, mortality (current study), and sex ratio (current study) of the beetle. Such a strong effect from the phorid fly, however, is obtained in combination with the ant. We propose that the seemingly lacking 1st degree HOI effect on beetle survival and sex ration is the result of two opposing forces associated with the parasitoid wasps and the ants. On the one hand parasitoid wasps are attracted to patches with ants, where they can find more and healthier beetle larvae. On the other hand, the ants in those patches behave aggressively toward the wasps offering some protection to the beetle larvae. Therefore, the pair-wise host-parasitoid interaction is insufficient to explain consumer-resource dynamics. Instead, expanding the study system to include one or more HOIs may help obtain a better understanding of complex food webs dynamics. In this study we did not add a negative control (phorids with no ants) because the phorids do not do anything when their host is not present. Furthermore, since in nature the phorid fly that parasitize the ants do not occur without its host, adding such negative control would have added little to the interpretation of what happens in nature.

These results suggest that the phorid fly could be responsible for enhancing the beetle’s (and perhaps the wasp) myrmecophily through eco-evolutionary dynamics. Since the phorid fly increases the parasitism rate of the beetle (Fig. 2), beetles able to use the ant would consequently have higher fitness than the counterparts that are unable to do so. On the other hand, being able to use the ant and the phorid-ant interaction increases the fitness of the wasp offspring, as the beetle hosts in the ant patches enjoy ant protection against other natural enemies and abundant food resources (the green coffee scale). Stronger phorid attacks on the ant (the strength of the 2nd degree HOI) may speed up the selection of the myrmecophilous trait in the beetle and perhaps in the wasp as well. Nevertheless, this single dataset would not be sufficient to answer the question of ‘whether the two interactive HOIs increase female beetles’ fitness’ as the reduced number of male beetles, at its extreme, might become a limiting factor to female beetles’ mating opportunity. From our previous study^21^, however, we learned that gravid female beetles were able to lay as many eggs in the presence of both HOIs as in the condition of no HOI (i.e.*,* free of the ant interference and the phorid attacks on the ants) and more eggs than when there is only one HOI (i.e.*,* ant interference). In the natural condition, the beetle larvae may also suffer from more predation in the condition of no ants. The situation of phorid attacks on the ants is one that the beetle is unable to avoid but adapt, as the phorids closely follow their *Azteca* ant hosts. There would be a certain cost of being associated with ants, in this case, increased male mortality of the beetle. The net effect of the phorid on the female beetles, however, might still be positive in a complex natural environment.

We expect that these multispecies and complex HOI structures are more ubiquitous than has been previously supported by the literature^16,19,40^. Cascading 2nd degree HOI interfering with the 1st degree HOI, as in our study, occurs across trophic levels in both terrestrial and aquatic ecosystems^18,19,40^. Although our system may seem idiosyncratic, the functional roles of the HOIs species are observable in other systems.

For example, the monarch butterfly suffers from the protozoic parasite (*Ophryocytis elektroscirrha*), and the cardenolides in the milkweed (*Asclepias curaassavica*) reduces the parasite infection of the butterfly. Yet, an aphid (*Aphis nerii*) alters the cardenolide concentration in the plant, increasing the infection rate of the butterfly^29^. Potentially, other organisms able to utilize the cardenolides for protection would experience similar dynamics in this type of interaction structure. On the evolutionary side, we know that the infected female monarch butterfly tend to lay eggs on anti-parasite milkweeds that reduce the transmission and the virulence of the parasite on the monarch caterpillars^41^. Such cross-generational medication would alter the intricacy of consumer-resource interaction between the butterfly and high vs. low-cardenolide milkweeds, an evolutionary consequence emerging from the 2nd degree HOI that a third-party herbivore (*A. nerii*) initiates. The habitat choices of infected vs. uninfected female butterflies might contribute to determining whether pair-wise density effects are sufficient for explaining the tri-trophic milkweed-butterfly-protozoic parasite system.

Similar effects have also been found in an arthropod-ant-bird system in pine canopies where predatory birds interrupt the mutualism between ants and ant-tended aphids^40^. Birds here play a similar functional role as the phorid fly in our system. By changing the foraging behavior of ants, birds reduce the ant-tended aphid and herbivore abundances. A later study in the same system found that birds (the 2nd degree HOI) but not ants (the 1st degree HOI) have an emergent effect on plant phytochemistry. Such an emerging effect is only revealed while ants are present^42^.

This study contributes to the emerging literature of empirical studies of cascading 1st and 2nd order HOIs and demonstrate that these interactions affect important population parameters (survival and sex ratio) of an organism at the base of the cascade (in this case the coccinellid beetle; Fig. 1). We argue that these HOIs can have important evolutionary consequences for the organisms involved; in this case, strengthening myrmecophilic traits in coccinellid beetle. We also argue that cascading HOIs and its evolutionary consequences are more prevalent in nature than previously recognized. Predicting the occurrences and the functions of higher-order interactions have been a challenge in ecology^16,28^. Systems such as ours, Lefèvre et al*.*^43^, and Mooney^40,42^ would contribute to exploring the pathways and demographic parameters in modeling the ecological and evolutionary effects of higher-order interactions^1,11,44^. From these studies, we learn that organisms at various trophic levels can be the initiators and receivers of such complex interactions, and non-feeding parameters are often involved. From a practical perspective, because the beetle in the study is a voracious predator of scale insects in coffee agroecosystems, understanding how these HOIs affect the survival and the sex ratio of the beetle is important for maintaining pest control services in these agroecosystems.

## Supplementary Information

Supplementary Information 1.

Supplementary Information 2.

Supplementary Information 3.

Supplementary Information 4.

## Data Availability

All data generated or analysed during this study are included in this published article and its supplementary information files.
