# Supplementary material for: Surprising effects of cascading higher order interactions
Source: Sci Rep. 2022 Nov 12;12:19378. doi: 10.1038/s41598-022-23763-z (PMC9653485; doi:10.1038/s41598-022-23763-z)
Supplement: Supplementary file 4 — Supplementary Information 4. [file 41598_2022_23763_MOESM4_ESM.pdf]

Supplementary Materials

Figure 1S.

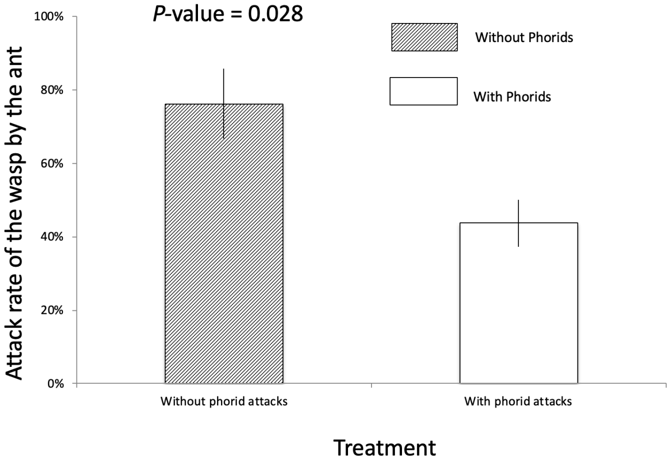

Figure 1S. The response of an individual ant to a parasitoid wasp (*Homalotylus shuvakhinae*) in the presence and absence of phorid flies (Mean ± S.E). *P*-value = 0.028

Field Survey of the phorid attack intensity and beetle sex and abundance

In 2009 and 2012, we sampled the phorid attack intensity and the beetle sex, in a 45-hectare permanent plot. In the two years, respectively, 56 and 36 sites of Azteca nest were selected, each of which is separated from another in a distance of at least 100m. At each site on each farm, we smashed a few ant workers in the bowl and placed the bowl at chest height at the tree. This action released the ant pheromones attracting the phorid fly parasitoids (*1*) which attacked *Azteca* ant workers. We then recorded the attack intensity of the fly on the ant by counting the frequency of the attacks in one minute. To sex the beetle, we collected all adults of *A. orbigera* spotted within 5 minutes on the four coffee bushes closest to a tree with an *Azteca* nest and sexed the adult beetles based on head color (females have blackheads and males have

orange heads). The survey was conducted roughly monthly from July to December, the rainy season when the organisms are the most abundant. The Maximum likelihood method was applied to estimate the beetle's sex ratio of each survey assuming a binomial distribution.

1. K. A. Mathis, S. M. Philpott, R. F. Moreira, Parasite lost: Chemical and visual cues used by Pseudacteon in search of Azteca instabilis. *Journal of Insect Behavior* **24**, 186-199 (2011).
